# Radiation Effects on Mortality from Solid Cancers Other than Lung, Liver, and Bone Cancer in the Mayak Worker Cohort: 1948–2008

**DOI:** 10.1371/journal.pone.0117784

**Published:** 2015-02-26

**Authors:** Mikhail Sokolnikov, Dale Preston, Ethel Gilbert, Sara Schonfeld, Nina Koshurnikova

**Affiliations:** 1 Laboratory of Epidemiology, Southern Urals Biophysics Institute, Ozyorsk Russia; 2 Hirosoft International, Eureka, California, United States of America; 3 Radiation Epidemiology Branch, Division of Cancer Epidemiology and Genetics, National Cancer Institute, Bethesda, Maryland, United States of America; 4 Section of Environment and Radiation, International Agency for Research on Cancer, Lyon France; Kagoshima University Graduate School of Medical and Dental Sciences, JAPAN

## Abstract

Radiation effects on mortality from solid cancers other than lung, liver, and bone cancer in the Mayak worker cohort: 1948–2008. The cohort of Mayak Production Association (PA) workers in Russia offers a unique opportunity to study the effects of prolonged low dose rate external gamma exposures and exposure to plutonium in a working age population. We examined radiation effects on the risk of mortality from solid cancers excluding sites of primary plutonium deposition (lung, liver, and bone surface) among 25,757 workers who were first employed in 1948–1982. During the period 1948–2008, there were 1,825 deaths from cancers other than lung, liver and bone. Using colon dose as a representative external dose, a linear dose response model described the data well. The excess relative risk per Gray for external gamma exposure was 0.16 (95% CI: 0.07 – 0.26) when unadjusted for plutonium exposure and 0.12 (95% CI 0.03 – 0.21) when adjusted for plutonium dose and monitoring status. There was no significant effect modification by sex or attained age. Plutonium exposure was not significantly associated with the group of cancers analyzed after adjusting for monitoring status. Site-specific risks were uncertainly estimated but positive for 13 of the 15 sites evaluated with a statistically significant estimate only for esophageal cancer. Comparison with estimates based on the acute exposures in atomic bomb survivors suggests that the excess relative risk per Gray for prolonged external exposure in Mayak workers may be lower than that for acute exposure but, given the uncertainties, the possibility of equal effects cannot be dismissed.

## Introduction

The Mayak Production Association (Mayak PA) in Ozyorsk, Russia, Chelyabinsk oblast, which began operation in 1948 in order to provide plutonium for the nascent Soviet nuclear weapons program, was the first Russian nuclear cycle enterprise. Mayak PA includes a reactor complex, radiochemical and plutonium production plants, and a number of auxiliary departments. Particularly during early years when the technology was still under development, Mayak workers in the main plants (reactor, radiochemical, and plutonium production) could receive substantial radiation exposures from external gamma-radiation and alpha-particles from incorporated plutonium (primarily ^239^Pu).

In the 1980’s and 90’s researchers at what is now called the Southern Urals Biophysics Institute (SUBI) established and began active follow-up of a Mayak Workers Cohort (MWC) that included all men and women who had ever worked in one of the main (reactor, radiochemical and plutonium production) plants or either of two auxiliary departments (water treatment and mechanical repair plants). As the cohort was created, efforts to develop individual external and internal dose estimates for the cohort members began. With almost 60 years of follow-up and individual dose estimates, the MWC provides a unique opportunity to study the effects of both internal Pu and prolonged low dose rate external gamma exposures in a working age population of men and women. The cohort has been used to describe radiation effects on cancer risks including studies of internal and external dose on cancer at the sites of primary Pu deposition (lung, liver, and bone surface) [1–4] and other solid cancer sites and leukemia [5,6] as well as some non-cancer health effects [7–9]. In this paper we consider radiation effects on the risk of solid cancer mortality at sites other than lung, liver, or bone surface since these three sites receive substantial doses from incorporated Pu while for other sites it is known that there is little or no internal exposure arising from Pu [10–12]. Results reported in this paper include 10 more years of follow-up than were used in the previous published analyses of these outcomes [5] and make use of improved internal and external dose estimates computed using the Mayak Worker Dosimetry System developed in 2008 (MWDS-2008) [10]. A paper on lung cancer risks, with a special emphasis on the effects of Pu exposure, has recently been published [13]. Papers on radiation effects on mortality rates for liver cancer, bone cancer, and leukemia and other hematopoietic malignancies in the cohort based on MWDS-2008 individual dose estimates and the current follow-up are being prepared separately.

## Materials and Methods

### Cohort definition

The MWC was defined in terms of occupational history obtained from Mayak personnel department records. The cohort includes all workers who started their employment in the period 1948–1982 at any one of the three main Mayak PA plants or at either the mechanical repair or water treatment plants. The MWC includes 25,757 people of whom about 25% are women.

### Occupational radiation exposure

Workers at the five facilities differed in terms of their (potential) exposures. Within any of the facilities exposures and doses have decreased markedly over time. Although they have higher average doses than those in many other cohorts with occupational radiation exposures [14], workers employed in the auxiliary plants had almost no potential for plutonium intake and much less potential for external exposure that cohort members who worked in one or more of the main plants. Reactor workers had greater potential for external exposure with no risk of Pu exposure. Radiochemical and plutonium production plant workers could receive external exposures and had the potential for plutonium intake. Within these plants the potential for plutonium intake decreased over time and the nature of the exposure depends on the technology in use and the types of Pu compounds at specific work places. Thus, for descriptive purposes we have subdivided the workplaces within the Pu production plant into three groups which are, in order of increasing potential for Pu exposure and dose: auxiliary departments; departments that we call “Main 2” where workers dealt primarily with Pu compounds of high transportability, and departments which we call “Main 1” where workers dealt primarily with Pu compounds of low transportability. For additional details concerning lung clearance of “low” and “high” transportability Pu compounds see [15–18]. Over the course of their careers, workers could work at more than one Mayak facility. For descriptive purposes we have classified workers according to their “primary workplace” defined as the plant/workplace in which a worker had the highest potential for Pu exposure throughout his/her occupational history until the beginning of 1983 (Table 1).

**Table 1:** General description of the Mayak Worker Cohort (MWC) by gender, workplace and period of first hire.

	Workers	Exposure charachteristics			Follow-up			
		External exposure	Plutonium		Deaths	Unknown cause (%)	Lost to follow-up (%)††	Lost to follow-up before 2004 (%)††
		Mean external dose† (mGy)	Monitored for Pu	Mean liver dose† (mGy)				
**Total**	25,757	354	7,059	266	12,438	0.6%	22.9%	4.9%
**Sex**								
Males	19,395	366	4,937	207	9,916	0.7%	22.9%	5.1%
Females	6,362	317	2,122	403	2,522	0.2%	22.9%	4.2%
**Primary work place**								
Auxiliary departments	3,384	76	55	16	1,386	0.9%	26.7%	5.9%
Reactor	5,416	334	307	41	2,700	0.7%	21.5%	4.7%
Radiochemical	9,194	601	3,683	203	4,586	0.6%	23.5%	5.4%
Plutonium Auxiliary	3,505	114	1,130	162	1,574	0.4%	23.2%	5.5%
Plutonium Main 2 (High Transportability)	1,994	155	920	103	952	0.2%	16.1%	1.8%
Plutonium Main 1 (Low Transportability)	2,264	359	964	870	1,240	0.7%	23.5%	3.7%
**Period of hire**								
1948–53	9,213	690	2,181	685	5,968	0.2%	25.4%	8.4%
1954–58	4,221	349	1,228	168	2,207	0.6%	27.9%	5.1%
1959–63	4,378	162	1,256	91	2,128	1.0%	28.0%	3.9%
1964–72	3,675	88	1,209	35	1,352	1.8%	16.8%	1.5%
1973–82	4,270	59	1,185	15	783	1.2%	12.3%	1.1%

† 5-year lagged cumulative colon dose at end of follow-up. External dose means include those with zero external exposure. Internal dose means are based on workers with urine bioassay-based dose estimates only.

†† Percentage of total number of workers.

### Follow-up

The methods of follow-up in MWC are described elsewhere [5,19]. The current analyses utilize follow-up through 2008. Follow-up starts at the time of first employment in an eligible plant and continues through the date of loss to follow-up, death, migration or censoring (as discussed below), or December 31, 2008, whichever occurred first. Cohort members who no longer live in Ozyorsk are considered to be migrants as of the date they moved from the city. Prior to 2004 it was possible to ascertain vital status and cause of death for most migrants but over the past 10 years privacy regulations have made it difficult to determine their vital status or cause of death. Therefore, all cohort members who had migrated and not died prior to January 1, 2004 are censored on December 31, 2003 and all cohort members migrating after this date are treated as lost-to follow-up as of their migration date. Cohort members not known to have migrated or died but whose date of last known vital status is prior to the December 31, 2008 are also treated as lost to follow-up after date of last known status. About 23% (5,893) of the cohort members are currently lost to follow-up including 4,497 migrants for whom follow-up was censored at the end of 2003, 132 who migrated in the period 2004–2008, and 1,264 people who were lost to follow-up before 2004 (Table 1).

### Ascertainment of vital status and registration of cause of death

SUBI staff engaged in active follow-up of vital status and cause of death for the cohort. Prior to 2004 regional address bureaus that record information about addresses and migration of all Russian citizens served as the primary source of information on current vital status and migration. As noted above, since 2004 we have not had routine access to address bureau information outside the city of Ozyorsk. However, it has remained possible to obtain virtually complete information on current vital status and cause of death information for cohort members who die or continue to reside in Ozyorsk as well as date of migration from Ozyorsk.

Among 12,438 deaths in the cohort members who were not lost to follow-up, underlying cause of death was determined from death certificates held at the Office of Civil Registry/Vital Statistics (ZAGS) for 57% of the deaths, from autopsy data for 21% of the deaths, from forensic autopsies for 12% of the deaths, and from relatives (often based on death certificates) for about 9% of the deaths (primarily for migrants). Cause of death was unknown for less than 1% of the deaths. Cause of death was coded using International Classification of Disease, Ninth Revision (ICD9) [20] and grouped into broader categories defined on the basis of the cause of death recodes (available at http://seer.cancer.gov/codrecode/) defined by the Surveillance, Epidemiology, and End Results (SEER) program of the US National Cancer Institute.

### Dosimetry

The current analyses are based on the 2008 version of the Mayak Worker dosimetry system (MWDS-2008). MWDS-2008 provides estimates of doses to specific organs from external sources for all workers based on either annual individual badge readings measurements (*measured badge reading*) or badge dose estimates based on modeled badge dose readings taking into account individual work histories (*reconstructed badge reading*). While 80% of the annual badge dose estimates are based on measured badge readings, 70% of the cohort members have at least one year in which dose estimates were based on reconstructed badge readings.

About 15% of the workers were reported to have had some occupational exposure to neutrons. The MWDS20008 system provides limited information on neutron doses. These dose estimates were not used in the current analyses. Future revisions of the dosimetry will include more complete information on neutron doses.

A small proportion of workers received acute exposures as a consequence of accidents or special incidents. While doses from such events account for much of the dose for some individual cohort members, they account for only a small fraction of the population dose. External gamma doses resulting from such exposures are accounted for by MWDS2008 and hence in the doses used in these analyses, but no additional efforts were made to allow for separating the effect of acute and chronic exposures.

Given an (measured or reconstructed) annual badge dose reading, annual personal dose equivalent Hp(10) and estimates of equivalent dose resulted from external gamma-exposure for 13 organs were computed using methods similar to those developed for the 2005 Mayak worker dosimetry system (*MWDS-2005*) described in [21]. These computations involve standardization of the adjustment for various exposure scenarios based on workplace and occupation, adjustment for non-routine exposures, corrections for the effects of high energy beta exposures on badge dose readings, and scenario-specific conversion from badge reading to the doses of interest. External dose estimates based on the MWDS-2008 differ from those in MWDS-2005 due to the development of additional exposure scenarios (including scenarios for water treatment and mechanical repair plant workers for whom doses were not computed previously).

The primary analyses of all solid cancers other than lung, liver, and bone as a group were carried out using the estimated colon dose resulting from exposure to external gamma-irradiation as a representative organ dose. Colon dose is similar to the doses to most internal organs. In addition, since colon dose is used for analyses of all solid cancers in the life span study cohort of atomic bomb survivors [22,23], its use in these analyses facilitates comparison of the external dose risk estimates in the Mayak workers with those seen in atomic bomb survivors. Furthermore, external colon doses in this cohort are similar to doses received by other gastro-intestinal organs and after exclusion of lung, liver and bone cancers, gastrointestinal cancers contributed about 50% of cancer deaths analyzed.

The cancers of interest in these analyses occurred in organs that are likely to have received little if any dose as a consequence of internal exposures arising from the inhalation or ingestion of plutonium or other alpha-emitting radionuclides, such as ^241^Am or ^238^Pu. Although we currently think that there is little potential for internal exposures other than ^239^Pu, future improvements of the dosimetry should provide more information on internal exposures arising from other radionuclides. In many of the analyses presented below we adjusted for possible effects of internal exposure on the external dose risk estimates. These adjustments were based on individual urine-bioassay-based time-dependent estimates of lagged cumulative liver dose arising from plutonium exposure when available and period- and workplace-specific categories of workers’ potential plutonium exposure for periods prior to monitoring. Monitoring data and hence internal dose estimates are available for about 40% of the radiochemical and plutonium plant workers. The MWDS-2008 systemic Pu biokinetic model [12] assumes that internal doses to the organs of interest for this report are essentially proportional to, but much orders of magnitude smaller than the dose to the liver.

Some details concerning the MWDS-2008 internal dose estimates are given in [13]. Briefly, mathematical models were developed to use the available bioassay data to produce annual activity and dose estimates for the lung, liver and bone surface. These methods make use of information on individual occupational histories, workplace-specific data on the physiochemical form of the plutonium aerosols in various workplaces, body mass, and smoking status. Information on smoking status was obtained from polyclinic medical records archived at SUBI. As described in the discussion of statistical methods, when bioassay-based internal dose estimates were not available the internal exposure adjustment was based on plutonium surrogate categories. These categories, which depend on time period and workplace, are described in Table 2. This is the same definition used in recent papers on lung, liver, and bone cancer risks in the cohort [3,13].

**Table 2:** Definition and selected characteristics of plutonium exposure surrogate categories.

Category	Definition		People	Monitored for Pu exposure (%)	Mean Dose†	
	Workplace	Period of hire			External colon (mGy)	Internal liver (mGy)
0	Reactor complex or Auxiliary plants	1948–1982	9,293	3.3%	222	43
1	Radiochemical or any Plutonium	1964–1982	4,891	49.4%	77	26
2	Radiochemical or Plutonium auxiliary	1954–1963	5,931	39.9%	324	105
	or Plutonium Main 2	1959–1963				
3	Radiochemical or Plutonium auxiliary	1948–1953	5,329	35.5%	810	419
	or Plutonium Main 2	1950–1958				
	or Plutonium Main 1	1959–1963				
4	Plutonium Main 2	1948–1949	740	36.4%	319	476
	or Plutonium Main 1	1954–1958				
5	Plutonium Main 1	1948–1953	573	37.0%	914	3,170
**Total**			**25,757**	**27.4**	**354**	**460**

† 5-year lagged cumulative dose at end of follow-up. Internal dose means based on workers with urine bioassay-based dose estimates.

In order to assess the impact of the change in dosimetry we present results of some analyses based on the external “dose” estimates used in our previous analysis of these data [5]. External dose estimates used in [5] were actually film badge readings transcribed from Mayak records by staff of the SUBI Laboratory of Epidemiology. These archive film badge readings, known as MWDS-2000, were used without adjustment for badge characteristics, workplace conditions, or the effect of changes in the units in which the doses were originally recorded, Roentgen (R) from 1948–1973 and rem from 1974 through the end of 1997. MWDS-2000 did not include any plutonium dose estimates, internal exposure adjustments in [5] were based on plutonium body burden estimates, a poor surrogate for dose from plutonium.

### Organization of data for analysis

For the primary analyses the data were structured as tables of person-years and case counts. These tables were stratified on sex, period of hire (1948–52,1953–57,1958–62,1963–72,1973–82), birth cohort (pre-1938, 1939–1942, 1943–1952, and 1953–1965), smoking status (ever, never, unknown), and plant. The table also included stratification on the time dependent variables attained age (5-year categories from ages 10 to 84 and 85 or more), calendar year(1948–49, five year categories form 1950 to 2003, 2004–07, and 2008), duration of follow-up (with cutpoints at 2, 5, 10, and 15 years), migration from Ozyorsk (resident, migrant), plutonium monitoring status (monitored, unmonitored) and time since monitoring (unmonitored, 0–1 years since monitored, 2 or more years since monitored), 5-year-lagged cumulative internal (liver) and external doses (colon) with a zero-dose category and non-zero-dose categories with boundaries at 10, 25,40,100,150,250, 500, 1000, 1500, 2000, 3000, and, for internal dose, 5000 mGy, and plutonium surrogate categories (6 categories defined by work history and time period). Each cell includes information on the number of person years at risk, the number of cases for each outcome of interest, person-year weighted average values of age, calendar time, external and internal dose and other continuous variables of interest. Because of indications that some workers were monitored for plutonium exposure as a result of suspected disease, workers were classified as unmonitored until two years after the first plutonium monitoring date.

For the most analyses, doses were lagged for five years, but we also created tables with alternative lags (2, 10, 15, and 20 years) to explore heterogeneity in the dose-response estimates. Analyses of cancers at specific sites were based on person-year tables that were stratified on external dose to the most relevant organ. Person year tables were created using the DATAB module of Epicure [24].

### Statistical Methods

Analyses were carried out using Poisson regression methods for generalized risk models implemented with the AMFIT module of Epicure [24]. Radiation effects on cause specific death rates were modeled using excess relative risk (ERR) models of the form λ_0_(*s,a,x*)[1+*ERR*]. In this model λ_0_(*s,a,x*) describes the baseline rates in terms of age (*a*), sex (*s*) and other factors including birth cohort trend, and smoking status (ever, never, unknown by sex). In some analyses, a time-dependent indicator of plutonium monitoring status was included in the baseline rate model when estimating or adjusting for internal exposure effects. As noted above, there were indications that some people were selected for monitoring as a result of their health status. This factor is only relevant and thus only included in models that adjusted for internal dose or plutonium-surrogate-category effects. Baseline rate models in the site-specific analyses always included attained age and sex effects with other variables when there were indications of significant effects.

The ERR was typically described as the sum of three components:

$$ERR=ERR_{ext}+I_{mon}ERR_{\mathrm{int}}+I_{unmon}ERR_{pusur}$$ (1)

where *ERR_ext_* and *ERR*_int_ describe, respectively, the effects of external and internal dose and *ERR_pusur_* describes the (possibly sex-dependent) excess relative risks associated with plutonium surrogate categories. *I_mon_* and *I_unmon_* are time-dependent 0/1 indicators of whether or not people are considered to have been monitored for plutonium exposure (by definition *I_mon_* = 1 - *I_unmon_*).

The ERR function describes the magnitude of the radiation-associated excess risk relative to the baseline as a function of external and internal dose and, possibly, other factors such as sex, age at exposure and time since exposure. Most analyses described in this report involve a simple linear ERR model for the external dose effects but we also used some non-linear dose response models. These models included a linear-quadratic and pure-quadratic dose response models (*β*_1_ *d*+*β*_2_ *d* ^2^ and *β*_2_ *d* ^2^, respectively), a linear cell-killing model in which the dose response could flatten at high dose (*β*_1_ *de* ^-*β*_2_d^), and a linear-threshold model (*β*_1_(*d*-*c*)^+^ where (*d*-*c*)^+^ is 0 if dose is less than the threshold value (*c*) and *d*-*c* for larger doses.

Attained age effects on the radiation ERR were analyzed using a model of the form *βde^αlog(age)^* = *βda^α^* in which the variation in the ERR is proportional to attained age to the power *α*. In order to examine time-since- or age-at-exposure effects on the external dose ERR we considered models of the form $ERR_{ext}=\sum_{i}\beta_{i}d(p_{i})$ where *d*(*p_i_*) is the dose accumulated in a particular period of interest. These analyses made use of specially constructed person-year tables. For time-since-exposure, the categories are defined in terms of years prior to the age at risk, for example the dose accumulated 2 to 4 years prior to the time at risk, 5 to 9 years, 10 to 14 years and so on. For age at exposure effects, the categories are defined in terms of the ages at which the dose was received, for example, prior to age 20, between 20 and 29, and so on.

Parameter estimates were obtained using maximum likelihood methods [25]. Hypothesis tests were carried out using likelihood ratio tests and confidence intervals (CI) were based directly on differences in the profile log-likelihood function [25]. The reported significance levels are based on two-sided tests.

## Results

Table 1 provides information about exposure characteristics and current follow-up status stratified by sex, primary workplace, and period of hire. Table 3 provides more detail on the distribution of workers and mean doses by sex, period of hire, and primary workplace. External doses were generally similar for males and females but varied substantially by period of hire and primary workplace. There were 12,438 deaths, including 2,980 cancer deaths (662 in women) during 950,896 years of follow-up. There were a total of 1,825 solid cancers other than lung, liver and bone.

**Table 3:** Number of workers and mean doses by sex, period of hire and primary plant.

	Period of Hire														
	1948–53			1954–58			1959–63			1964–72			1973–82		
	People	Ext. dose†	Int. dose‡	People	Ext. dose	Int. dose	People	Ext. dose	Int. dose	People	Ext. dose	Int. dose	People	Ext. dose	Int. dose
**Auxilary plants**															
**Male**	523	171	115	310	97	32	440	56	4	717	55	15	719	36	5
**Female**	184	146	—	47	50	—	114	70	7	183	37	1	147	27	4
**Reactor complex**															
**Male**	1,768	621	47	575	360	34	643	239	40	481	179	70	771	99	6
**Female**	676	226	83	151	105	28	70	70	24	78	69	14	203	50	9
**Radiochemical plant**															
**Male**	2,333	1,196	532	1,682	519	126	1,229	278	88	683	152	35	916	82	25
**Female**	1,327	867	332	272	442	94	233	144	39	187	104	31	332	62	12
**Plutonium auxiliary departments**															
**Male**	666	279	253	209	150	62	431	47	75	468	34	33	454	21	7
**Female**	531	214	713	100	84	227	202	23	128	184	12	27	260	22	11
**Plutonium high transportability departments (Main 2)**															
**Male**	353	379	288	287	133	141	414	97	82	274	90	25	262	54	10
**Female**	112	315	286	45	152	234	71	62	41	93	63	31	83	63	8
**Plutonium low transportability departments (Main 1)**															
**Male**	453	792	1,477	457	270	376	492	141	165	297	50	55	88	46	15
**Female**	287	766	3,736	86	202	368	39	106	95	30	22	47	35	21	15

† Mean 5-year lagged cumulative colon dose at end of follow-up, mGy

‡ Mean 5-year lagged cumulative internal liver dose in mGy at end of follow-up for workers with urine bioassay-based internal dose estimates

— No workers monitored for plutonium exposure.

### Background mortality for solid cancers other than lung, liver and bone as a group

**Fig 1:**
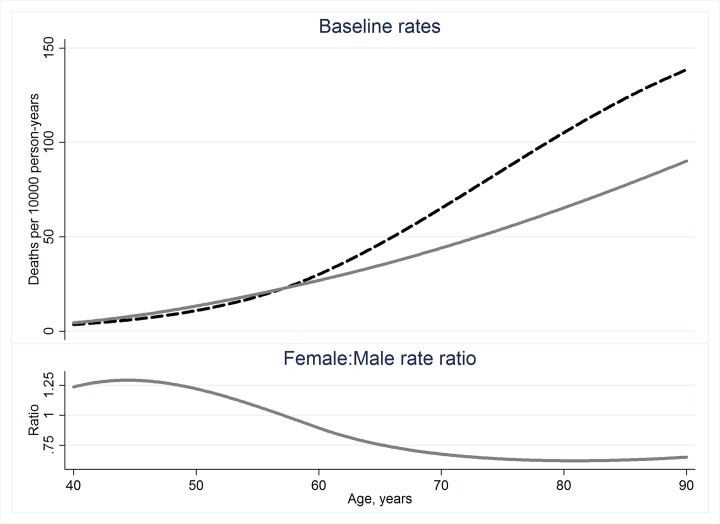
Non-smoker baseline cancer mortality rates and their sex ratio. Non-smoker baseline rates for mortality from solid cancers in organs other than lung, liver, bone or connective tissue in the Mayak worker cohort (upper panel) with age-specific sex ratios (lower panel).

For the combination of all solid cancers other than lung, liver and bone, the increase in the baseline was roughly proportional to attained age to the fifth power but with a slightly less rapid increase for women than for men. Although the sex-difference in the baseline rates depends somewhat on age (with women having slightly higher rates prior to about age 60 and much lower rates later in life), on average age-specific baseline rates for women were about 80% (95% CI 70% to 94%, P = 0.006) of those for men. The plots in Fig. 1 illustrate the age-dependence and sex-ratio of the baseline rates for never smokers. Baseline rates for ever-smokers for this group of solid cancers were estimated to be about 50% greater than those for never-smokers (95% CI 34% to 74%, P < 0.001). There was no indication of a significant sex-difference in the smoking effect (P > 0.5). As reported earlier, background cancer mortality after migration was about 15% lower than mortality while residing in Ozyorsk [5].

### Radiation risk estimates

**Fig 2:**
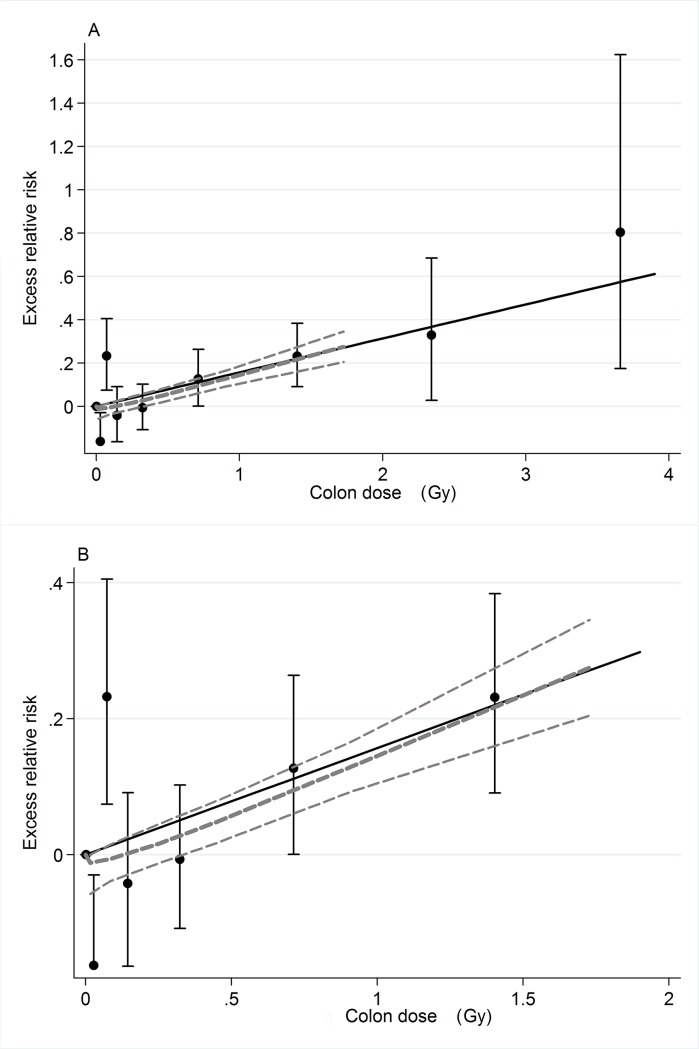
External exposure dose response for solid cancers other than lung liver and bone. A) External exposure dose response function for solid cancers at sites of than lung, live, bone, or connective tissue. B) The same plot for doses below 1.5 Gy. The solid line is the fitted linear dose response, the points are ERR estimates in dose categories. The thick dashed line is a non-parametric smooth fit to the categorical estimates while the thin dashed lines indicate plus or minus one standard error from the smoothed curve. The models used in this analysis included no adjustment for plutonium exposure.

**External dose**. Using a linear dose response model with adjustment for internal Pu exposure and Pu monitoring status we found a statistically significant (P = 0.01) external dose response for colon dose for solid cancers in organs other than lung, liver and bone. The ERR was estimated as 0.12 per Gy (95% CI 0.03 to 0.21). With this model we estimated that about 97 of the 1,825 deaths from these cancers were associated with the external exposure. The ERR per Gy for external dose slightly increased to 0.13 (95% CI 0.05–0.23) when adjusting for Pu exposure but not monitoring status. Without any adjustment for internal exposure or plutonium monitoring status, the external dose ERR/Gy estimate increased to 0.16 (95% CI 0.07 to 0.26, P < 0.001) and the estimated number of external-exposure-associated cases was 128. The distributions of the estimated numbers of background and excess cases over dose categories with and without adjustment for internal exposure effects are given in Table 4. Fig. 2 Illustrates the fitted dose response together with dose-category–specific ERR estimates and a non-parametric smoothed fit based on these estimates.

**Table 4:** Observed and fitted solid cancer deaths excluding lung, liver, bone, and connective tissue cancer deaths by external dose category with and without adjustment for internal exposure.

External colon dose (Gy)	Person year	Deaths	Internal-exposure adjusted †			Unadjusted ‡	
			Background	Exposure-associated		Background	External dose associated
				External	Internal		
<0.01	277,422	246	219.6	0.04	0.2	222.6	0.1
-0.05	140,927	168	200.9	0.7	0.2	200.6	0.9
-0.10	98,648	213	174.6	1.5	0.5	172.9	2.0
-0.2	106,818	216	226.9	3.8	0.7	225.5	5.2
-0.5	141,808	342	348.4	13.1	1.9	344.3	17.7
-1	93,270	282	252.2	20.7	2.2	250.2	28.0
-2	72,944	271	228.4	37.4	4.3	220.1	48.9
-3	15,146	63	50.7	13.8	1.8	47.4	17.5
3+	3,913	24	13.7	5.8	1.0	13.3	7.7
**Total**	**950,894**	**1,825**	**1,715.4**	**96.9**	**12.7**	**1,697.1**	**127.9**

† The baseline risk model includes an adjustment for the time-dependent Pu monitoring status and the excess relative risk includes internal dose and surrogate category effects for monitored and unmonitored workers, respectively.

‡ The baseline risk model does not include a Pu monitoring effect and there are no internal dose or surrogate effects in the ERR.

Consideration of linear-quadratic or models with cell-killing effects at higher doses did not provide any indication of significant non-linearity in the dose response with P > 0.5 for any of the models considered) (Table 5). While the maximum likelihood estimate of a threshold is about 0.2 Gy (95% CI: 0 to 1.3), the profile likelihood was virtually constant for thresholds between 0 and 0.75 Gy.

**Table 5:** External dose effect estimates from selected excess relative risk (ERR) models for solid cancers other than lung, liver, and bone cancers.

Model/Parameter description	Estimate (95% CI*)	
**5-year lagged cumulative dose time-constant ERR**		
***Linear dose response***		
Adjusted for internal exposure (ERR/Gy)	0.12	(0.03 to 0.21)
No internal exposure adjustment (ERR/Gy)	0.16	(0.07 to 0.26)
**Linear-Quadratic dose response† (P > 0.5)**		
Linear (ERR/Gy)	0.10	(-0.09 to 0.30)
Quadratic (per Gy^2^)	0.03	(-0.05 to 0.11)
**Linear- dose response with cell killing† (P > 0.5)**		
Linear (ERR/Gy)	0.11	(-0.05 to 0.30)
Cell-Killing	0.02	(-0.05 to 0.08)
**Time Since Exposure windows† (P**_**het**_ * > 0.5)		
***Years since dose received***	**ERR/Gy**	
*5–9*	0.44	(<0 to 1.5)
*10–14*	-0.08	(<-0.2 to 0.56)
*15–19*	0.15	(<0 to 0.62)
*20 ++*	0.16	(0.07 to 0.27)
**Age at exposure windows† (P**_**het**_ **> 0.5)**		
***Ages (years) dose received***	**ERR/Gy**	
< 25	0.14	(-0.005 to 0.30)
25–29	0.28	(0.08 to 0.51)
30–39	0.06	(0.004 to 0.25)
40–49	0.15	(-0.09 to 0.47)
50+	0.06	(<-0.1 to 1.1)

* 95% confidence interval

† Estimates made without adjustment for internal exposure. P-values are for likelihood ratio tests comparing model shown to linear time constant ERR model in lagged 5 cumulative dose. P_het_ refers to the P-value for a test for heterogeneity of risk estimates over categories of a variable of interest.

There was no evidence that the linear dose-response effect for external exposure differed by sex (P > 0.5) or migration status (P > 0.5). The best estimate of the female to male ERR sex ratio was 1.0 with a 95% CI of -0.13 to 1.6 (P > 0.5). The negative lower bound arises because the ERR for women was not significantly greater than 0. There was no indication of significant variability in the ERR with attained age. In particular, when the ERR was allowed to vary in proportion to attained age to a power, the best estimate of the power was -0.11 (which implies that the risk was nearly constant) with a 95% confidence interval ranging from -3.1 to 4.0 (P>0.5). There was no evidence of significant heterogeneity in the ERR for doses received 5 to 9, 10 to 14, 15 to 19, or 20+ years prior to death (P > 0.5, Table 5). Also, while there was no indication of significantly elevated risks associated with doses received 2 to 4 years prior to death (ERR/Sv 0.15, 95% CI < -0.2 to 1.4, P > 0.5), the risk in this window was similar to the ERR of 0.16 for doses received 5 or more years prior to death. Analyses of variation in the ERR with the age at which doses were received provided no evidence that this variable modified risks (P > 0.5) (Table 5). Inferences about external dose effect modification were similar when risks were adjusted for internal exposure.

**Internal Exposure effects for solid cancers other than lung, liver, and bone**. Risk increased with increasing internal dose to the liver (P < 0.006) in a model that used data for the full cohort with plutonium surrogate-category adjustments. However, when baseline rates were adjusted for monitoring status age-specific death rates were 16% higher (95% CI 11% to 39%, P < 0.001) for monitored than for unmonitored workers. Furthermore, after making this adjustment, the estimated plutonium ERR per unit liver dose was less than half that in the unadjusted analysis and was no longer statistically significant (P = 0.14). In view of the elevated baseline rates for monitored workers with plutonium monitoring data and since adjustment for monitoring status had such a marked effect on the estimated plutonium dose response parameter and its statistical significance, we focus on external dose effect estimates and our site-specific analyses of cancers other than lung, liver, and bone were unadjusted for internal exposure.

**Site-specific risk estimates**. Table 6 summarizes the distribution of deaths by site and sex for the cancers of interest for this paper as well as for solid cancers at sites of primary plutonium deposition (lung, liver, and bone) and for leukemia and other lympho-hematopoietic malignancies. Among men, lung (33%) and stomach (16%) were the most common cancer sites while breast (16%), lung (12%), and stomach (12%) were the most common sites among women.

**Table 6:** Cancer cases by sex and site.

Type of cancer	Men	Women	Total
**Tissues with limited plutonium deposition**			
Stomach	374	78	452
Colon	110	46	156
Rectum	103	43	146
Pancreas	103	25	128
Breast		107	107
Prostate	80		80
Kidney	61	17	78
Melanoma	23	15	38
Larynx	66	2	68
Esophagus	58	8	66
Brain & central nervous system	58	8	66
Bladder	62	1	63
Ovary		43	43
Uterus		36	36
Other solid cancer	236	65	301
*Group total*	*1334*	*491*	*1825*
**Tissues with major plutonium deposition**			
Lung	760	81	841
Liver	61	30	91
Bone and soft tissue	28	14	42
*Group total*	*849*	*125*	*974*
**Lympho-hematopoietic malignancies**			
Leukemia	82	28	110
Other lympho-hematopoietic	53	18	71
Lympho-hematopoietic total	135	46	181
**Total**	**2318**	**662**	**2980**

In analyses of site-specific cancers, baseline rates were adjusted for sex, age and smoking. For those sites with small numbers of deaths among women (bladder and larynx) analyses were limited to men and deaths from these cancers among women were included in the remainder group. The primary results, which are presented in Table 7, were based on analyses that used the relevant organ dose estimates without adjustment for plutonium exposure effects. We also carried out site-specific analyses with adjustment for plutonium exposure with results available online. There was no indication that adjustment for plutonium effects greatly modified the external exposure results shown in Table 7.

**Table 7:** Site-specific excess relative risk per Gy estimates‡ and number of external exposure associated cases.

Site	Deaths	Dose	Linear risk estimate			Radiation-associated cases
			ERR	95% CI	P	
Colon	156	Colon	0.21	-0.06–0.62	0.13	14.1
Esophagus	66	Esophagus	1.26	0.36–3.27	< 0.001	24.7
Stomach	452	Stomach	0.12	-0.03–0.31	0.06	25.8
Rectum	146	Rectum	0.18	-0.09–0.63	0.18	11.7
Pancreas	128	Pancreas	0.18	-0.09–0.64	0.18	11.6
Bladder*	62	Bladder	-0.02	<-0–0.45	>0.5	-0.8
Kidney	78	Kidney	0.08	<-0.1–0.69	> 0.5	2.8
Melanoma	38	Hp(10)	-0.00	<-0.1–0.77	> 0.5	0.0
Brain	66	Brain	< 0	< -0.1 0.32	0.50	-3.2
Larynx*	66	Larynx	0.12	<0–0.68	>0.5	4.7
Prostate	80	Prostate	0.11	<0–0.63	>0.5	5.0
Breast	107	Breast	0.16	-0.09–0.57	>0.5	7.3
Ovary	43	Ovary	0.19	<0–1.23	> 0.5	2.7
Uterus	36	Uterus	0.42	<0–1.66	0.23	4.4
Remainder	301	Colon	0.09	-0.05–0.30	0.24	16.7
Total excluding lung, liver bone	1825	Site-specific organ doses				127.5
		Colon	0.16	0.07–0.26	< 0.001	127.2
		Film badge	0.08	0.03–0.14	<0.01	98.2

‡ The risk estimates in this table were not adjusted for plutonium exposure since, as noted in the text, there is no evidence of significant effects of plutonium for all of these cancers as a group or for any specific type of cancer considered here.

* Analyses were limited to cases seen in men with cases among women (1 bladder cancer and 2 laryngeal cancers) included in the remainder.

Although the point estimates of the external exposure ERR per Gy for all sites except bladder and brain were positive, a statistically significant (P < 0.001) external dose effect was observed only for esophagus (ERR per Gy 1.3 with a 95% CI of 0.36 to 3.3). The total number of fitted external radiation-exposure-associated cases was virtually identical to that seen for analyses of the combined category of all solid cancers other than lung, liver and bone based on colon dose (127)

**Film badge doses**. Previously we reported analyses based on film badge doses adjusted for internal exposure using a combination of estimated plutonium body burden for monitored workers and plutonium surrogate categories for unmonitored workers [5]. The linear ERR/Gy estimate in [5], which was adjusted for Pu exposure, was 0.08 (95% CI 0.03 to 0.21). However there was some indication of downward curvature in the dose response and the low dose slope in a linear quadratic model fit to the earlier data was 0.21 (95% CI 0.06 to 0.37). Current analyses differ from [5] in terms of the extended follow-up (through the end of 2008 instead of the end of 1997 in [5]), use of improved external and Pu doses (MWDS-2008) and adjustment for smoking status in the background rates. To compare risk estimates obtained from the different dosimetry systems we repeated the calculations above using film badge external doses instead of colon dose estimates. Using the same follow-up (through the end of 2008), estimates of risk related to film badge dose followed the same pattern as risk estimates based on colon dose. ERR per Gy for external film badge dose was lower (0.08, 95% CI 0.03–0.14) than the ERR estimate calculated using colon dose (0.16, 95% CI 0.07–0.26), which would be expected since colon dose estimates are lower than film badge doses. The overall mean colon dose in this cohort was 354 mGy (Table 1), the overall mean film badge dose is 510 mGy. The linear-quadratic model yielded similar results using the film badge doses with a linear coefficient of 0.12 and curvature coefficient of -0.02 (see Table 5 for comparison). However when follow-up was restricted to the end of 1997 the risk coefficients were much closer to those observed in previous analyses with a linear-quadratic model providing a somewhat improved fit to the data (P<0.08).

## Discussion

There is considerable interest in the effects of prolonged, low-dose-rate occupational radiation exposures on the risks of cancer and other diseases. With high-quality, long-term follow-up and the relatively large doses received by many workers, the Mayak worker cohort is a major source of information on such risks. Because many cohort members received significant exposures resulting from inhalation of plutonium aerosols, the analyses presented here have focused on dose effects for solid cancers in tissues other than those that are the primary sites for plutonium deposition (i.e., lung, liver, and bone), which were considered in earlier papers [3,13].

We found a clear statistically significant increase in the risk of all solid cancers other than lung, liver, and bone with increasing external occupational gamma dose. Risks for this group of solid cancers were estimated to be increased by about 16% per Gy of external gamma dose (95% CI 7% to 26%) without adjustment for Pu exposure and 12% per Gy (95% CI 3% to 21%) with such adjustment. Largely because of the small number of cases for specific cancer types, this study has limited power to detect statistically significant increased risks at specific sites. It should be noted however, that point estimates of the radiation effect were positive for all but two (bladder and brain/CNS tumors) of the 15 sites and groups of sites considered here.

These analyses update earlier results for the cohort [5] by adding 10 additional years of follow-up, extending the cohort under study to include workers hired in 1973–1982. They also make use of newly developed organ dose estimates (in place of the film badge dose readings) and of improved information on internal Pu exposure. The primary reason for differences in the risk estimates from the current and earlier analyses is the shift from film badge readings to colon dose estimates. This change resulted in increased risk estimates and reduced evidence for non-linearity in the dose response.

Risk estimates from the Life Span Study (LSS) of Japanese atomic bomb survivors have long been the primary source of the cancer risk estimates that are used as the basis for recommended radiation protection standards [26,27]. One of the most important issues in radiation protection concerns the applicability of risk estimates derived from acute high dose rate exposure to atomic bomb radiation to the prolonged low-dose rate exposures that are of primary concern in most occupational and environmental exposure scenarios. Because of the magnitude of the doses, the Mayak worker cohort is one of the most informative occupational cohorts for characterization of the effects of prolonged, low dose-rate radiation exposure on cancer risks. Table 8 presents risk estimates, mean doses, and other information from our current analyses along with similar information from the LSS [28], the 15-country nuclear worker study [29], the United Kingdom’s National Registry of Radiation Workers [30] and the Techa River cohort [31]. The LSS risk estimates were based on analyses of publicly available data obtained from RERF (http://www.rerf.jp/library/dl_e/lss13.html) and were restricted to survivors who were between 20 and 60 years of age at the time of exposure with deaths limited to those from solid cancers other than lung, liver, bone and connective tissue. In order to account for the marked difference in the sex distribution of the two cohorts (75% of Mayak workers were men while men constitute only 33% of the working age LSS members), we estimated a gender-averaged risk in the LSS with weights of 0.75 for men and 0.25 for women.

**Table 8:** Excess relative risk (ERR) estimates from the A-bomb survivors and from selected studies of persons exposed to protracted low-LET external ionizing radiation.

Study population	Mean dose	Outcome	Deaths		ERR Per Gy(Sv)	
					**Est.**	**90% CI**†
Mayak nuclear workers	354	Solid cancers except lung, liver, and bone	Men:	1,334	0.15	(0.06–0.27)
			Women:	491	0.17	(0.02–0.35)
			Total:	1,825	0.16	(0.08–0.24)
A-bomb survivors exposed between 20 and 60 [28]	109	Solid cancers except lung, liver, and bone	Men:	2,278	0.30	(0.10–0.54)
			Women:	3,088	0.52	(0.32–0.74)
			Total:	5,366	0.35††	(0.19–0.55)
Nuclear workers in 15 countries [29]§ §	19	All except leukemia, lung, and pleura		3,390‡	0.44	(-0.30–1.4)
United Kingdom National Registry for Radiation Workers (NRRW) [30]	25	All except leukemia, lung, and pleura		5,118‡	0.32	(0.02–0.67)
Techa River Cohort [31]	35	All except leukemia		2,303	0.61**	(0.13–1.2)

* Doses are external colon dose in mGy for Mayak workers, weighted colon dose in mGy for the atomic bomb survivors, Hp(10) in mSv for the 15-country and NRRW studies.(Hp(10) is the equivalent dose at a tissue depth of 10 mm beneath a dosimeter); stomach dose in mGy for the Techa river cohort

† 90% confidence intervals used for comparison with published results in the 15-country and NRRW studies

†† weighted average with weights of 0.75 for men and 0.25 for women to reflect the sex ratio in the Mayak worker cohort

‡ These populations are predominantly male

§ The estimate presented here is based on the 15-country study results withwith the Canadian data excluded ([29], page 405).). This estimate was used because of concerns about the Canadian data usedused in that study. This concerns have beenbeen supported by the recently published re-analysis of the Canadian workerworker data given in [34]. The ERR/SvSv estimate for using all of the 15-country data is 0.59 (95% CI <0 to 1.5, [29], page 403).

** There was no evidence that risk differed by sex in this cohort.

Among the studies included in Table 8, the Mayak worker cohort has the smallest risk estimates and is the only study for which the estimated ERR is less than that seen in the LSS. In view of the uncertainties for the risk estimates shown in the table, there is no compelling evidence that the external dose effects in the Mayak worker cohort differ from those seen in the other studies. A simple test for heterogeneity gives p-value of 0.11 between Mayak and the A-bomb results.

Recently, Hunter et al. (6) evaluated cancer incidence in the Mayak worker cohort. Their analyses included 1,447 incident cases of solid cancers other than lung, liver, and bone that were diagnosed in Ozyorsk in the period 1948–2004. Using a statistical approach that was similar to ours, the estimated ERR/Gy was 0.07 (95% CI: 0.01to 0.15) without adjustment for Pu exposure, and slightly lower when such adjustment was added. This estimate is smaller than our mortality-based estimates partly because it was based on *H*p(10) (equivalent dose at a tissue depth of 10 mm beneath a dosimeter) instead of colon dose. When we repeated our analyses using *H*p(10) (see S1 Table provided in Supplementary material), our estimate of 0.16, based on colon dose and unadjusted for Pu exposure, was reduced to 0.12 (95% CI: 0.05 to 0.20), closer to but still larger than the estimate in (6). Differences in the estimates based on incidence and mortality are likely explained primarily by the differences in the group of cancers analyzed. The 1,447 cancer cases evaluated in [6] included cancer cases that had not resulted in death and thus were not included in our analyses. The 1,825 deaths in our analyses included cancer deaths occurring in migrants, in the period 2005–2008, and in auxiliary plant workers, none of which would have been included in [6] unless the cancer was diagnosed in Ozyorsk before the end of 2004.

Our analyses are subject to limitations caused by using information on cause of death from death certificates and autopsy findings. Although this information was obtained primarily from death certificates, autopsy findings were used when available and for 9% of the deaths the cause was coded based on information obtained from relatives. The agreement between death-certificate and autopsy-based cause of death information for Ozyorsk residents was examined in [19] and found to be quite good, but some misclassification can’t be ruled out. Another limitation is the loss of power resulting from our inability to follow migrants in the last 10 years due to privacy protection issues. In addition, despite the large overall number of solid cancer deaths, estimates of site-specific cancer risks are imprecise [32]. Estimation of solid cancer risk resulting from prolonged occupational external gamma-exposure is complicated by the fact that radiochemical and Pu production plant workers were potentially exposed to Pu-containing aerosols. We have provided external dose risk estimates that are adjusted for Pu exposure, but this adjustment is limited by several factors: 1) only about 40% of the workers with potential for Pu exposure have urine-bioassay data; 2) routine monitoring did not begin until many years after the largest exposures and appears to have depended on health status; 3) Pu dose estimation from bioassay data is complicated and the resulting estimates have large uncertainties; and 4) the Pu-exposure-potential categories used prior to bioassay-based dose estimates imprecisely characterize Pu exposures [10–12,21,33]. The uncertainties and limitations of the Pu dose and surrogate categories could result in some bias in the external dose estimates.

Because of the above difficulties, we have given only limited attention to risks from Pu exposure. We note, however, that while there was some evidence of a Pu dose effect on the risk of solid cancers other than lung, liver, and bone, further analyses suggested that this effect may have been largely the result of factors related to the selection of subjects for Pu monitoring. In addition, given the very small internal doses to organs other than lung, liver and bone, statistical power for detecting risks from these doses is limited. Another difficulty is that the Pu exposure-response relationship might be biased by misclassified cancers of the lung, liver, or bone.

The strengths of this study include the high quality follow-up of Ozyorsk residents throughout the study period and of migrants prior to 2004 as well as the availability of individually-measured external radiation doses and detailed occupational histories. The dose range resulting from the prolonged occupational exposures of the Mayak workers is broader and the mean doses higher than in other worker cohorts.

There is currently little information on factors such as smoking and alcohol consumption that could affect the baseline rates and could be confounded with radiation dose. We were able to adjust the baseline rates for smoking status (ever, never, unknown) and found that this had little effect on the radiation risk estimates. Unfortunately, although efforts are being made to extract improved information on alcohol consumption from medical records for individual workers, the alcohol consumption data available at this time is rather crude and not adequate for use in the risk modeling.

While there is considerable interest in site-specific cancer risk estimates from the Mayak worker cohort, because of the relatively small number of cancers for specific sites, the power to detect radiation-associated site-specific risks is limited. In the current analyses, the only site with a significant effect was esophageal cancer. The ERR/Gy estimate for this site was the largest of all sites considered and is about twice the incidence ERR estimate seen in the atomic bomb survivors [22,23]. This may be a chance finding or it could reflect effect modification or confounding by some unmeasured risk factor. One possibility is alcohol consumption, which is a well-known risk factor for cancer of the esophagus. However, it seems unlikely that confounding with alcohol would greatly distort the radiation risk estimate since the limited data on alcohol consumption data suggest that there is little correlation between alcohol consumption and radiation dose. Since alcohol consumption levels are much greater in Russia than in Japan, the difference between the Russian and Japanese radiation ERR estimates could arise if there is a large super-multiplicative interaction between radiation and alcohol. At present it is impossible to judge if this is the explanation for the difference that was seen, but as more information on alcohol consumption becomes available, it may be possible to learn more about the joint effect of radiation and alcohol consumption.

Due to privacy protections and other issues, cohort follow-up is becoming increasingly difficult. As noted earlier, since 2004 it has not been possible to obtain vital status and cause of death information for migrants and as a result these people are treated as lost to follow-up from 2004 for survivor cohort members who migrated before that date and the migration date for more recent migrants. This reduces the power of the study especially in later years. The relatively large proportion (about 9%) of deaths for which the cause was determined from sources other than a death certificate or autopsy report is also a limitation. However, some (as yet informal) comparison of the reports from close relatives as the cause of death compared to the death-certificate- or autopsy-based causes of death suggest that these reports are reasonably accurate at the level of detail used in these analyses.

The results of these analyses of the Mayak worker cohort clearly demonstrate increased cancer risks following prolonged low dose rate radiation exposure. While the ERR point estimate in this study is about half of that seen in a comparable subset of the atomic bomb survivors the uncertainties in these estimates are such that one may not rule out equal effects of acute and prolonged exposures. Further analyses of the Mayak worker cohort with additional follow-up, improved dose estimates, and more information on non-radiation risk factors will result in improved characterization of the effects low-dose rate prolonged exposures.

## Supporting Information

S1 Table Site-specific risk estimates calculated using Hp(10) dose. (DOCX)

S1 Script Person-years tabulation script to be used with Epicure software. (ZIP)

## References

[1] Gilbert ES, Koshurnikova NA, Sokolnikov M, Khokhryakov VF, Miller S, Preston DL, et al Liver cancers in Mayak workers. Radiat Res. 2000; 154: 246–252. 10956429 10.1667/0033-7587(2000)154[0246:lcimw]2.0.co;2

[2] Gilbert ES, Koshurnikova NA, Sokolnikov ME, Shilnikova NS, Preston DL, Ron E, et al Lung cancer in Mayak workers. Radiat Res. 2004; 162: 505–516. 15624305 10.1667/rr3259

[3] Sokolnikov ME, Gilbert ES, Preston DL, Ron E, Shilnikova NS, Khokhryakov VV, et al Lung, liver and bone cancer mortality in Mayak workers. Int J Cancer. 2008; 123: 905–911. 10.1002/ijc.23581 18528867 PMC4020282

[4] Labutina EV, Kuznetsova IS, Hunter N, Harrison J, Koshurnikova NA. Radiation risk of malignant neoplasms in organs of main deposition for plutonium in the cohort of mayak workers with regard to histological types. Health Phys. 2013; 105: 165–176. 10.1097/HP.0b013e31828f57df 23799501

[5] Shilnikova NS, Preston DL, Ron E, Gilbert ES, Vassilenko EK, Romanov SA, et al Cancer mortality risk among workers at the Mayak nuclear complex. Radiat Res. 2003; 159: 787–798. 12751962 10.1667/0033-7587(2003)159[0787:cmrawa]2.0.co;2

[6] Hunter N, Kuznetsova IS, Labutina EV, Harrison JD. Solid cancer incidence other than lung, liver and bone in Mayak workers: 1948–2004. Br J Cancer. 2013; 109: 1989–1996. 10.1038/bjc.2013.543 24022197 PMC3790189

[7] Azizova TV, Zhuntova GV, Haylock RG, Moseeva MB, Grigoryeva ES, Hunter N, et al Chronic bronchitis in the cohort of Mayak workers first employed 1948–1958. Radiat Res. 2013; 180: 610–621. 10.1667/RR13228.1 24219326

[8] Azizova TV, Muirhead CR, Druzhinina MB, Grigoryeva ES, Vlasenko EV, Sumina MV, et al Cardiovascular diseases in the cohort of workers first employed at Mayak PA in 1948–1958. Radiat Res. 2010; 174: 155–168. 10.1667/RR1789.1 20681782

[9] Azizova TV, Muirhead CR, Druzhinina MB, Grigoryeva ES, Vlasenko EV, Sumina MV, et al Cerebrovascular diseases in the cohort of workers first employed at Mayak PA in 1948–1958. Radiat Res. 2010; 174: 851–864. 10.1667/RR1928.1 21128809

[10] Khokhryakov VV, Khokhryakov VF, Suslova KG, Vostrotin VV, Vvedensky VE, Sokolova AB, et al Mayak Worker Dosimetry System 2008 (MWDS-2008): assessment of internal dose from measurement results of plutonium activity in urine. Health Phys. 2013; 104: 366–378. 10.1097/HP.0b013e31827dbf60 23439140

[11] Romanov SA, Guilmette RA, Khokhryakov VF, Phipps A, Aladova EE, Bertelli L, et al Comparison of dose estimation from occupational exposure to 239Pu using different modelling approaches. Radiat Prot Dosimetry. 2007; 127: 486–490. 18045798 10.1093/rpd/ncm415

[12] Leggett RW, Eckerman KF, Khokhryakov VF, Suslova KG, Krahenbuhl MP, Miller SC. Mayak worker study: an improved biokinetic model for reconstructing doses from internally deposited plutonium. Radiat Res. 2005; 164: 111–122. 16038582 10.1667/rr3371

[13] Gilbert ES, Sokolnikov ME, Preston DL, Schonfeld SJ, Schadilov AE, Vasilenko EK, et al Lung cancer risks from plutonium: an updated analysis of data from the Mayak worker cohort. Radiat Res. 2013; 179: 332–342. 10.1667/RR3054.1 23391147 PMC3661277

[14] Cardis E, Vrijheid M, Blettner M, Gilbert E, Hakama M, Hill C, et al Risk of cancer after low doses of ionising radiation: retrospective cohort study in 15 countries. Bmj. 2005; 331: 77 15987704 10.1136/bmj.38499.599861.E0 PMC558612

[15] Khokhryakov VF, Suslova KG, Kudryavtseva TI, Schadilov AE, Vostrotin VV, Lagounova NY, et al Relative role of plutonium excretion with urine and feces from human body. Health Phys. 2004; 86: 523–527. 15083148 10.1097/00004032-200405000-00009

[16] Khokhryakov VF, Suslova KG, Vostrotin VV, Romanov SA, Menshikh ZS, Kudryavtseva TI, et al The development of the plutonium lung clearance model for exposure estimation of the Mayak production association, nuclear plant workers. Health Phys. 2002; 82: 425–431. 11906131 10.1097/00004032-200204000-00001

[17] Khokhryakov VF, Romanov SA, Suslova KG. A model of lung clearance of plutonium In: Van Kaick G, Karaoglou A, Kellerer AM, editors. Health effects of internally deposited radionuclides: Emphasis on radium and thorium: World Scientific Co. Pte. Ltd 1995; pp. 117–121.

[18] Khokhryakov VF, Menshikh ZS, Suslova KG, Kudryavtseva KG, Tokarskaya ZB, Romanov SA. Plutonium excretion model for the healthy man. Radiation Protection and Dosimetry. 1994; 53: 235–239.

[19] Koshurnikova NA, Shilnikova NS, Okatenko PV, Kreslov VV, Bolotnikova MG, Sokolnikov ME, et al Characteristics of the cohort of workers at the Mayak nuclear complex. Radiat Res. 1999; 152: 352–363. 10477912

[20] World Health Organization. Manual of the international statistical classification of diseases, injuries, and causes of death: based on the recommendations of the Ninth Revision Conference, 1975, and adopted by the Twenty-ninth World Health Assembly. Geneva, Albany, N.Y.: World Health Organization; obtainable from WHO Publications Centre. 1977.

[21] Vasilenko EK, Khokhryakov VF, Miller SC, Fix JJ, Eckerman K, Choe DO, et al Mayak worker dosimetry study: an overview. Health Phys. 2007; 93: 190–206. 17693770 10.1097/01.HP.0000266071.43137.0e

[22] Ozasa K, Shimizu Y, Suyama A, Kasagi F, Soda M, Grant EJ, et al Studies of the mortality of atomic bomb survivors, Report 14, 1950–2003: an overview of cancer and noncancer diseases. Radiat Res. 2012; 177: 229–243. 22171960 10.1667/rr2629.1

[23] Preston DL, Ron E, Tokuoka S, Funamoto S, Nishi N, Soda M, et al Solid cancer incidence in atomic bomb survivors: 1958–1998. Radiat Res. 2007; 168: 1–64. 17722996 10.1667/RR0763.1

[24] Preston DL, Lubin JH, Pierce DA, McConney ME Epicure Users Guide Seattle, Washington: Hirosoft International Corporation 1993.

[25] Cox DR, Hinkley DV Theoretical statistics London,: Chapman and Hall 1974.

[26] The 2007 Recommendations of the International Commission on Radiological Protection. ICRP publication 103. Ann ICRP. 2007; 37: 1–332. 10.1016/j.icrp.2007.10.003 18082557

[27] National Research Council (U.S.). Committee to Assess Health Risks from Exposure to Low Level of Ionizing Radiation Health risks from exposure to low levels of ionizing radiation: BEIR VII Phase 2. Washington, D.C.: National Academies Press 2006. 25077203

[28] Preston DL, Shimizu Y, Pierce DA, Suyama A, Mabuchi K. Studies of mortality of atomic bomb survivors. Report 13: Solid cancer and noncancer disease mortality: 1950–1997. Radiat Res. 2003; 160: 381–407. 12968934 10.1667/rr3049

[29] Cardis E, Vrijheid M, Blettner M, Gilbert E, Hakama M, Hill C, et al The 15-Country Collaborative Study of Cancer Risk among Radiation Workers in the Nuclear Industry: estimates of radiation-related cancer risks. RadiatRes. 2007; 167: 396–416. 17388693 10.1667/RR0553.1

[30] Muirhead CR, O'Hagan JA, Haylock RG, Phillipson MA, Willcock T, Berridge GL, et al Mortality and cancer incidence following occupational radiation exposure: third analysis of the National Registry for Radiation Workers. Br J Cancer. 2009; 100: 206–212. 10.1038/sj.bjc.6604825 19127272 PMC2634664

[31] Schonfeld SJ, Krestinina LY, Epifanova S, Degteva MO, Akleyev AV, Preston DL. Solid cancer mortality in the techa river cohort (1950–2007). Radiat Res. 2013; 179: 183–189. 10.1667/RR2932.1 23289384 PMC3613701

[32] Preston DL, Krestinina LY, Sokolnikov ME, Ron E, Davis FG, Ostroumova EV, et al How much can we say about site-specific cancer radiation risks? Radiat Res. 2010; 174: 816–824. 10.1667/RR2024.1 21128806

[33] Bess JD, Krahenbuhl MP, Miller SC, Slaughter DM, Khokhryakov VV, Khokhryakov VF, et al Uncertainties analysis for the plutonium dosimetry model, doses-2005, using Mayak bioassay data. Health Phys. 2007; 93: 207–219. 17693771 10.1097/01.HP.0000266741.42070.e8

[34] Zablotska LB, Lane RS, Thompson PA. A reanalysis of cancer mortality in Canadian nuclear workers (1956–1994) based on revised exposure and cohort data. Br J Cancer. 2014; 110: 214–223. 10.1038/bjc.2013.592 24231946 PMC3887280

