# Supplementary material for: Radiation Effects on Mortality from Solid Cancers Other than Lung, Liver, and Bone Cancer in the Mayak Worker Cohort: 1948–2008
Source: PLoS One. 2015 Feb 26;10(2):e0117784. doi: 10.1371/journal.pone.0117784 (PMC4342229; doi:10.1371/journal.pone.0117784)
Supplement: S1 Table — (DOCX) [file pone.0117784.s001.docx]

Table S1. Site-specific risk estimates calculated using Hp(10) dose.

|  |  | Unadjusted for Pu exposure | | | | Adjusted for Pu exposure | | | |
| --- | --- | --- | --- | --- | --- | --- | --- | --- | --- |
| Site | Deaths | Linear risk estimate | | | Excess cases due to external exposure | Linear risk estimate | | | Excess cases due to external exposure |
|  |  | ERR | 95% CI | P |  | ERR | 95% CI | P |  |
| Colon | 156 | 0.19 | -0.03 - 0.54 | 0.1 | 16.7 | 0.13 | -0.08 - 0.46 | >0.50 | 11.6 |
| Esophagus | 66 | 0.93 | 0.27 - 2.42 | <0.001 | 24.7 | 1.22 | 0.26 - 4.00 | 0.06 | 23.2 |
| Stomach | 452 | 0.10 | -0.03 - 0.27 | 0.16 | 24.2 | 0.09 | -0.05 - 0.26 | 0.36 | 21.1 |
| Rectum | 146 | 0.11 | <0 - 0.45 | 0.38 | 9.0 | 0.05 | <0 - 0.40 | >0.50 | 4.2 |
| Pancreas | 128 | 0.14 | -0.1 - 0.53 | 0.3 | 10.1 | 0.07 | <0 - 0.43 | 0.39 | 4.9 |
| Bladder* | 62 | -0.01 | <0 - 0.39 | > 0.50 | -0.5 | -0.01 | <0 - 0.44 | >0.50 | -0.6 |
| Kidney | 78 | 0.14 | <0 - 0.61 | 0.39 | 5.9 | 0.05 | <0 - 0.51 | >0.50 | 2.4 |
| Melanoma | 38 | 0.00 | <0 - 0.76 | >0.50 | -0.05 | 0.15 | <0 - 1.53 | 0.05 | 2.6 |
| Brain | 66 | <0 | <0 - 0.26 | >0.50 | <0 | <0 | - | - | - |
| Larynx* | 66 | 0.12 | <0 - 0.62 | 0.44 | 5.2 | 0.08 | <0 - 0.62 | 0.21 | 3.1 |
| Prostate | 80 | 0.08 | <0 - 0.51 | >0.50 | 4.3 | 0.04 | <0 - 0.46 | 0.50 | 2.4 |
| Breast | 107 | 0.16 | -0.09 - 0.58 | 0.27 | 7.2 | 0.09 | <0 - 0.55 | >0.50 | 3.9 |
| Ovary | 43 | 0.09 | <0 - 0.85 | > 0.50 | 1.8 | <0 | - | - | - |
| Uterus | 36 | 0.29 | <0 - 1.24 | 0.27 | 4.1 | 0.25 | <0 - 1.41 | >0.50 | 3.3 |
| Remainder | 301 | 0.09 | -0.06 - 0.29 | 0.26 | 16.3 | 0.07 | -0.07 - 0.27 | 0.30 | 12.6 |
| Total | 1825 |  |  |  | 129.0 |  |  |  | 94.7 |
|  |  | 0.12 | 0.05 - 0.20 | <0.001 | 126.5 | 0.09 | 0.02 – 0.16 | 0.35 | 107.4 |
